# Test-retest reliability and minimal detectable change of the Contextual Memory Test in older adults with and without mild cognitive impairment

**DOI:** 10.1371/journal.pone.0236654

**Published:** 2020-07-31

**Authors:** Wan-wen Liao, Ching-yi Wu, Chien-Hsiou Liu, Szu-hung Lin, Hui-Yan Chiau, Chia-ling Chen

**Affiliations:** 1 Department of Occupational Therapy, Graduate Institute of Behavioral Sciences, Chang Gung University, Taoyuan, Taiwan; 2 Healthy Aging Research Center, Chang Gung University, Taoyuan, Taiwan; 3 Department of Physical Medicine and Rehabilitation, Chang Gung Memorial Hospital, Linkou, Taiwan; 4 Department of Occupational Therapy, College of Medicine, Fu Jen Catholic University, New Taipei City, Taiwan; 5 Graduate Institute of Early Intervention, College of Medicine, Chang Gung University, Taoyuan, Taiwan; Nathan S Kline Institute, UNITED STATES

## Abstract

**Background:**

The ability to detect one’s own memory capacity and develop strategies based on daily contexts is important for daily activities. The Contextual Memory Test (CMT) assesses self-awareness, self-efficacy, self-perception/evaluation of performance, recall, and strategy use that are associated with daily contexts, and could be a potentially suitable measurement for assessing memory and meta-memory in older adults with and without cognitive impairment. Nevertheless, the test-retest reliability and minimal detectable change (MDC) remain unknown in these individuals.

**Objective:**

The purpose of this study was to examine test-retest reliability and calculate MDC of the CMT in healthy older adults and those with mild cognitive impairment (MCI).

**Methods:**

Eighty-three participants completed the CMT twice with a one-month interval. Test-retest reliability was examined using intraclass correlation coefficient (ICC) in all seven domains of the CMT and the recognition subtest. The standard error of measurement (SEM) and MDC were calculated. The Bland-Altman analysis was performed to evaluate the degree of agreement between measurements.

**Results:**

The ICC of five domains (self-awareness, self-perception/evaluation of performance, immediate/delayed/total recall) and the recognition subtest were good to excellent (ICC = 0.63–0.94) in healthy and MCI participants and the MDC% were less than 30% The ICC of the other two domains (self-efficacy and total strategy use, TSS) were low (ICC = 0.07–0.59) and the MDC% exceeded 30%. The Bland-Altman analysis showed generally better performance in the 2^nd^ than the 1^st^ measurement in most CMT domains.

**Conclusions:**

Our results revealed sufficient test-retest reliability and acceptable MDC in most CMT domains in healthy and MCI participants. Only the self-efficacy and TSS domains demonstrated low ICC and large MDC. Possible practice effects were found between repeated measurements. Clinicians should be cautious when evaluating self-efficacy and strategy use using the CMT in older adults. Further improvements are needed for these two domains.

## Introduction

Meta-memory refers to one’s own knowledge and awareness about his/her own memory performance [1]. It is an important underlying component of most activities of daily living [2, 3]. To perform various daily activities, individuals need to have good insights of their memory and predict whether they would be able to complete the desired tasks and if not, which strategies should be taken to successfully complete the desired tasks. These processes heavily relied on memory and meta-memory function including self-awareness, self-efficacy, and prediction/estimation of one’ own performance [1]. Nevertheless, memory and meta-memory often decline with advancing age [4]. Studies have demonstrated that older adults with subjective memory impairments (SCI) or mild cognitive impairment (MCI) had greater chances of transferring from SCI/MCI to dementia if they had impaired meta-memory function [5, 6]. Accurately assessing and monitoring memory and meta-memory changes in older adults is thus critical for early identification of at-risk individuals to prevent memory and meta-memory decline.

A variety of measurements have been developed to assess and monitor memory and meta-memory changes. Common memory assessments include the Wechsler Memory Scale [7] and Memory Assessment Scale [8]. Although these measurements provide information about memory function, they do not address implications of memory deficits on daily life. Furthermore, most memory assessments examined memory capacity using names, digits and texts (e.g., word-pairs) that do not involve activities of daily living [9]. As a result, outcomes of these measurements may not necessarily reflect actual performance in daily life.

Common meta-memory assessments such as the Memory Functioning Questionnaire (MFQ) [10] and Meta-memory in Adulthood (MIA) [11] primarily examined individuals’ subjective awareness/efficacy of memory, frequency of forgetting things and strategy use in basic and/or complex activities of daily living. However, one major drawback of these measurements was the large numbers of items in the questionnaires (e.g., 108 items in MIA and 64 items in MFQ) and the considerable amount of time to complete the tests. Although the short-form versions of these measurements have been developed, they tend to focus on only one or some domains of the original assessments (e.g., self-efficacy domain of MFQ) [12, 13]. Furthermore, despite evaluation of strategy use, most meta-memory assessments did not assess whether individuals could recognize daily living contexts and use context cues to develop strategies to remember/recall items involving daily activities. The ability to spontaneously recognize familiar daily contexts and develop according strategies is crucial for independent living [14, 15]. Understanding if older adults, particularly those with MCI, still retain this ability will facilitate design of appropriate treatment plans to prevent memory or meta-memory deterioration and improve independent living in the early phase of cognitive decline.

To address the above issues, the Contextual Memory Test (CMT) was primarily developed to assess memory and meta-memory related to daily contexts [16–18]. The CMT aims to determine if individuals can spontaneously detect daily contexts and use contextual strategies to remember/recall associated items, and if providing context cues will improve individuals’ memory and meta-memory performance related to daily contexts. The CMT was developed based on theoretical frameworks of memory processing including the stage model and the context-dependent theory [19]. The stage model hypothesizes that memory processing involves three different stages, which are encoding, storage and retrial and memory can be enhanced through manipulation of these three stages [19]. For example, presenting the item to be remembered within a particular context is likely to induce an association strategy between the items and the context, which may facilitate encoding and storage of memory. In this way, the context will be used as a cue or strategy to activate the stored memory, and subsequently enhancing recall of this item in the context. This context-dependent memory is highly involved in activities of daily living because most essential daily tasks, such as grooming, bathing, or dining are embedded in a particular daily scene. The purpose of the CMT is to assist healthcare professionals in identifying individuals that may have impaired context-related memory, for example the inability to recognize the context cue or the inability to use the contextual information as a strategy to recall items in everyday tasks [16]. Furthermore, the CMT can be used to assess context-related memory/meta-memory changes over time, which may help healthcare professionals to evaluate patients’ improvement after treatments [16].

The CMT consists of 16 questions and two picture cards. It has seven domains addressing memory and meta-memory function including (1) self-efficacy, (2) self-perception and evaluation of performance, (3) immediate recall, (4) delayed recall, (5) total recall, (6) total strategy use, and (7) general self-awareness, and an additional recognition subtest. Specifically the items to remember/recall are listed in the two picture cards and each card involves a common daily context, one is the morning theme and the other is the restaurant theme. For example, items that usually involve morning routines such as the tooth brush, comb and razor are listed in the morning picture card and items that usually involve in the restaurant context such as a waitress, receipts and menus are listed in the restaurant picture card.

The cross-culture validation, construct and discriminative validity of CMT have been examined in several previous studies. Josman et al. (2000) [18] evaluated the construct validity of the CMT to discriminate between three age groups (i.e., young, middle aged and older adults) and the applicability of the CMT in an Israeli population. They found significant differences in the CMT outcomes between these three groups, indicating good discriminative validity. In addition, this study also demonstrated that the CMT could be properly used in the Israeli participants. This finding further indicated the CMT may be appropriate for use in different population across regions. The discriminative validity of CMT has also been examined in healthy older adults and those with Alzheimer’s disease (AD) [17]. Significant differences were identified in the CMT outcomes between these two groups of participants, suggesting good discriminative validity.

Although the construct and discriminative validity have been examined, the reliability of the CMT, for example the test-retest reliability has not fully explored in healthy older adults and those with mild cognitive impairment. Previous studies primarily determined the reliability of CMT in individuals with neurological impairments including stroke, hematoma and head trauma [16], and they found good test-retest reliability in the recall, self-prediction of performance and total strategy use domains. A comprehensive evaluation of test-retest reliability including the relative reliability (i.e., intra-class correlation coefficient, ICC), absolute reliability (i.e., the standard error of measurement, SEM) and minimal detectable change (MDC) of all CMT domains and the agreement between repeated measurements are important pre-requisite for using the CMT as a screening tool for identifying individuals with potential memory and meta-memory impairment and an assessment tool for evaluating memory and meta-memory changes after treatments in these individuals.

The purpose of this study was to determine the test-retest reliability and the MDC of the CMT in older adults with and without MCI. The findings of this study can inform clinicians/researchers of the reproducibility and measurement errors of the CMT in healthy and MCI older adults and enhance usability of CMT in clinical/research settings.

## Materials and methods

### Participants

This study was a prospective trial with a repeated testing design. Participants were assessed by the CMT twice, with a one-month interval in between tests. Participants were enrolled from community centers, senior daycare centers, and retirement homes in northern Taiwan. The inclusion criteria were (1) age greater than 50 years old and (2) adequate global cognitive function to follow instruction (the Mini-Mental State Examination scores≥17). The exclusion criteria were (1) having hemi-neglect or visual agnosia, (2) depression symptoms (i.e., the Geriatric Depression Scale-short form ≥ 5) [20], (3) anxiety symptoms (the Geriatric Anxiety Inventory-short form (GAI-SF)-five items≥3 (GAI-SF score range: 1–5) [21], and (4) diagnosis of dementia (e.g., Alzheimer’s disease or vascular dementia). The Montreal cognitive assessment (MOCA) was used to identify participants with mild cognitive impairment. The one-point educational adjustment (addition) was applied for participants with less than 12 years of education [22]. Participants with MOCA scores ≥ 26 were classified as healthy participants and participants with MOCA scores ≥ 17 and < 26 were classified as MCI participants [22, 23]. All participants provided written informed consents and the study procedures were approved by the Institutional Review Board of Fu Jen catholic university, Taiwan. All study procedures followed the declaration of Helsinki.

### Procedures

Participants undertook the CMT twice, with a one-month interval. The raters administered the CMT following the procedures in the CMT manual [16]. The raters were trained by the principal investigators of this study to ensure they performed CMT correctly in a standardized manner. Participants’ demographics and clinical information was collected prior to the 1^st^ measurement.

### Measurements

#### Structures and procedures

**Fig 1:**
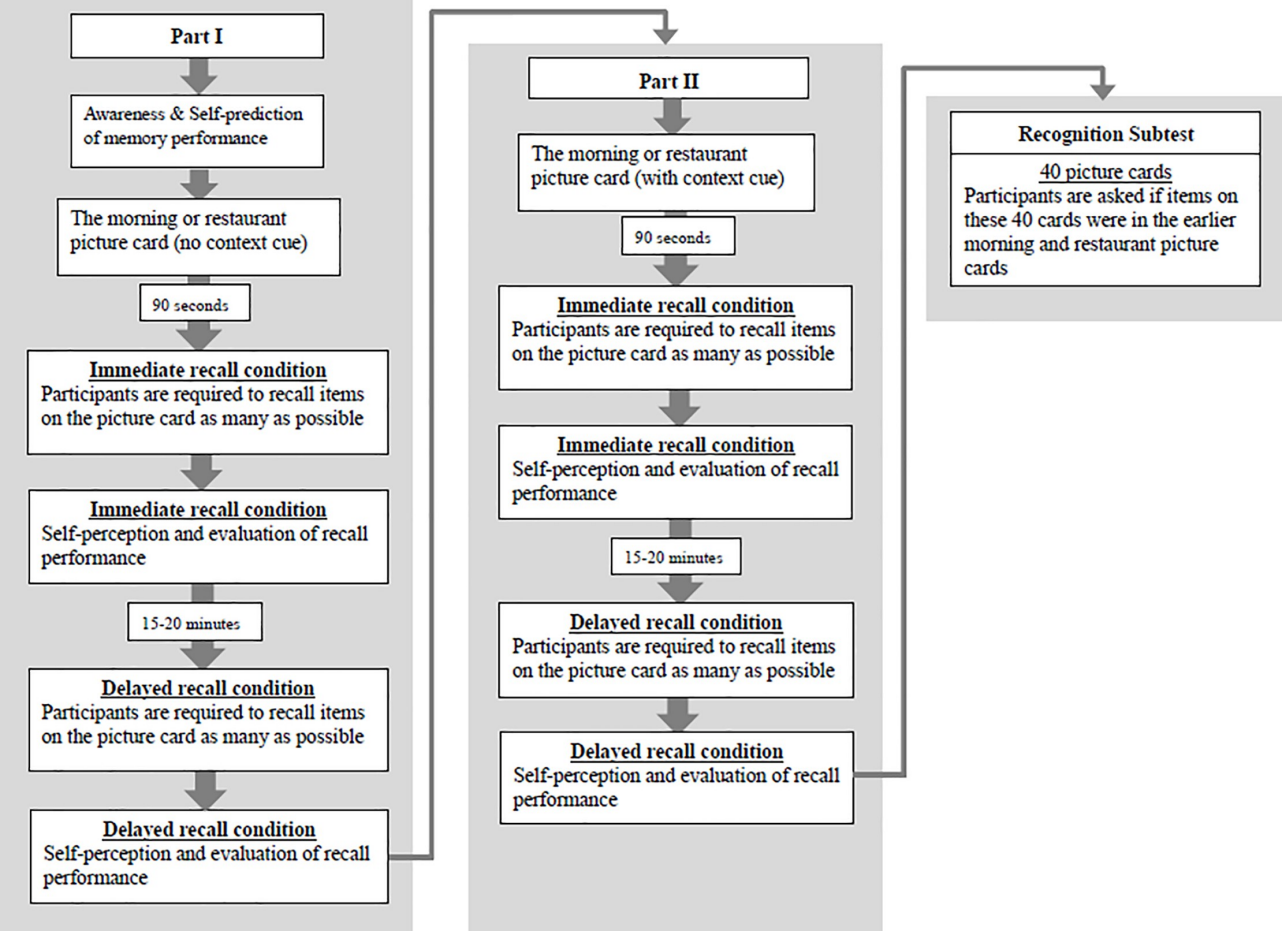
The structure and procedure of the CMT. The CMT have two parts (Part I and Part II) in the immediate and delayed recall conditions, and an additional recognition subtest. The immediate recall condition always starts first followed by the delayed recall condition. The order of the CMT is Part I (immediate recall), Part I (delayed recall), Part II (immediate recall), Part II (delayed recall), and the recognition subtest. The context cue was provided only in Part II based on the standard instruction on the CMT manual. Participants were asked to think of what a person usually does when getting up in the morning and getting ready to leave the house (morning version) or think of a restaurant scene and the sequences of events that occur when a person is at the restaurant (restaurant version).

The CMT consists of 16 questions and two picture cards [16]. Each picture card contains 20 line drawings of items related to the morning routines or the restaurant theme (i.e., the morning version and the restaurant version). This design allows evaluation of participants’ ability to recognize the daily context and use the context as a strategy to remember/recall items. Participants were asked to remember and recall the items on the picture cards as many as possible. Fig 1 shows the structure and procedure of the CMT.

Part I began by asking participants standard questions about their awareness of memory function (question 1, 2, and 5–9), and their ability to predict memory performance (question 3). Then, the morning or restaurant picture card was shown to the participants and 90 seconds were given for participants to remember the 20 items on the picture card. After 90 seconds, participants were asked to recall the items as many as possible (immediate recall). The evaluator wrote down the numbers of items correctly recalled by the participants. After completing the picture card test, participants were asked how they felt about the picture card test including the difficulty of the test and their performance during the test (question 10–12) and estimated the numbers of items they had recalled (question 13). In addition, the evaluator assessed if the participants used any strategies to remember/recall those 20 items (question 15–16). Higher strategy use scores indicated that participants could spontaneously recognize the daily context and used it as the primary strategy to remember/recall items.

After an interval of 15 to 20 minutes, participants were asked to recall the items on the same picture card as many as possible (delayed recall). The evaluator asked again about participants’ perception (question 10–12) and estimation of performance (question 13) in the delayed recall condition.

In Part II, participants were given information about the context of the picture card. The purpose of Part II was to examine if participants could utilize the context cue as the primary strategy to remember/recall associated items. Part II began with the picture card test and followed by self-perception and evaluation (question 10–12), estimation of memory performance (question 13) and evaluation of strategy use (question 15–16) in both immediate and delayed recall conditions.

Part I was always performed first followed by a one-to-two hour break, and then Part II was delivered. Either the morning or restaurant version was used in Part I or Part II. For example, if the morning picture card was used for Part I, the restaurant picture card would be used for Part II. In this study, the order of morning and restaurant versions was randomized for each participant. This order was kept consistent for each participant in the 1^st^ and 2^nd^ measurements.

If participants failed to perform Part I and Part II, an additional subtest, which was the recognition subtest, would be administered. In the recognition subtest, 40 object cards were presented one at a time to the participants. Participants were asked if the presented object was in the earlier picture card test. The evaluators recorded the numbers of objects correctly identified by the participants.

In this study, Part I, Part II and the recognition subtest were all performed to comprehensively examine the reliability and MDC of all CMT domains. Participants were assessed in a quiet room to minimize interferences that may affect their cognitive responses. They were also required to relax and rest in the 15–20 minutes break between immediate recall and delayed recall conditions and in the one-to-two hour break between Part I and Part II.

#### Scoring

The CMT was scored independently in seven domains, which were the (1) self-efficacy domain including the prediction discrepancy and estimation discrepancy, (2) self-perception and evaluation domain (i.e., perceives task as difficult), (3) immediate recall (IR) (i.e., the numbers of items correctly recalled during the immediate recall condition), (4) delayed recall (DR) (i.e., the numbers of items correctly recalled during the delayed recall condition), (5) total recall (TR) (i.e., the numbers of total items correctly recalled in both immediate and delayed recall conditions), (6) total strategy use (TSS), and (7) general self-awareness (GA), as well as the recognition subtest (morning and restaurant versions). All domains were scored in Part I and Part II except the GA and prediction discrepancy, which were administered once in Part I. The scoring procedures followed the instruction on the CMT manual [16].

### Statistical analysis

The ICC value represents the relative reliability. It was calculated based on the two-way random model and the absolute agreement (ICC_2,1_) [24]. An ICC value greater than 0.8 indicates excellent relative reliability; between 0.61 and 0.8 indicates good reliability; between 0.41 and 0.6 indicates moderate reliability and lower than 0.4 indicates poor reliability [25].

The SEM value represents the absolute reliability, which was the degree to which repeated measurements vary for individuals [26]. A smaller SEM indicates a better absolute reliability. The MDC is the minimal amounts of changes required to be considered as a real change and exceed the random errors [27]. The MDC_95_ was calculated in this study. It is a commonly used index that computed the MDC based on the 95% confidence interval (see the formula below). We calculated the percent of MDC_95_ (i.e., MDC%) because it was independent of the units of measurements. The MDC% was calculated by dividing the MDC_95_ by the maximum scores of each domain and multiplying it by 100 [28]. The MDC% less than 30 was considered an acceptable random measurement error based on recommendations from the literatures [28, 29]. The SEM and MDC_95_ were calculated based on the formula below,

$$MDC_{95}=z‐value\times SEM\times \surd 2$$

$$SEM=SD_{1}\times \surd (1-\mathrm{ICC})$$

where SD_1_ is the standard deviation of outcomes in the 1^st^ assessment. z-value is the 95% confidence interval of a normal distribution (i.e., 1.96).

In addition, we also performed the Bland-Altman analysis to examine the agreement between repeated measurements [30]. The 95% limits of agreement (LOA) were calculated for each CMT domain, which were the mean differences (MD) of the two measurements ± 1.96SD. The LOA indicates the possible range of mean differences between two measurements for most individuals. In addition, a one-sample-test was used to examine if the mean differences (MD) between two measurements significantly deviated from 0, which suggested potential systematic bias [31]. The MD was the scores of the 2^nd^ measurement subtracted the scores of the 1^st^ measurement. A positive value indicated higher scores in the 2^nd^ than the 1^st^ measurement.

We conducted a sample size calculation based on a desired reliability coefficient of 0.8 reported in the previous reliability studies [16–18] and a minimum acceptable coefficient of 0.6 [25]. With an alpha value of 0.05, a power of 0.8 and two testing sessions, a minimum sample size of 39 was required for each group [32]. In addition to the reliability tests, we also performed an independent t test to examine whether there were differences in the age and educational levels between the healthy and MCI groups.

## Results

Eighty-three participants were enrolled. All of them completed the two assessments. Among them, forty-four were healthy participants and thirty-nine were MCI participants. The mean age of healthy participants was 66.5 years old and their averaged MOCA scores were 28.14. The mean age of MCI participants was 69.44 years old and their averaged MOCA scores were 22.49. The age was similar between the two groups (*t* = -1.37, *P* = 0.18). The education level was higher in the healthy than MCI participants (*t* = 4.48, *P*<0.001). Table 1 shows the demographics and clinical characteristics of all participants.

**Table 1:** Demographics and clinical characteristics of healthy and MCI participants (N = 83).

Characteristic	Healthy (n = 44)	MCI (n = 39)
Age (years)	66.5 (8.99)	69.44 (10.56)
Gender (male/female), n	9/35	13/26
Education (years)	14 (3.44)	9.58 (5.25)
GDS	0.75 (1.06)	1.38 (1.35)
GAI	0.45 (0.9)	0.33 (0.77)
MOCA	28.14 (1.41)	22.49 (2.84)
MMSE	29.5 (0.73)	27.49 (2.44)

Value is presented as mean(standard deviation); MCI, Mild cognitive impairment; GDS, Geriatric Depression Scale; GAI, Geriatric Anxiety Inventory-Short Form; MOCA, Montreal Cognitive Assessment; MMSE, Mini-Mental State Examination.

Table 2 summarizes the ICC, SEM and MDC values of all CMT domains in Part I, Part II and the recognition subtest in healthy participants. Most ICC values were between 0.63 and 0.92, indicating good to excellent reliability except the self-efficacy (estimation discrepancy, Part I and II) and TSS (Part II) domains. The ICC values of the self-efficacy domain (estimation discrepancy, Part I and II) and TSS domain (Part II) were below 0.6 (range: 0.24–0.59). The SEM values were between 0.34 and 2.75. Only the MDC% values of the self-efficacy domain (prediction discrepancy) and TSS domain (Part II) were above 30% (32.22% and 35.24%).

**Table 2:** Results of the test-retest reliability and MDC of the CMT in healthy participants (n = 44).

	Part I. No context cue			Part II. Context cue		
	ICC (95% CI)	SEM	MDC_95_ (MDC%)	ICC (95% CI)	SEM	MDC_95_ (MDC%)
Self-efficacy						
Prediction discrepancy	0.71 (0.47, 0.84)	2.32	6.44 (32.22%)*			
Estimation discrepancy-IR	0.29* (-0.31, 0.61)	1.98	5.48 (27.4%)	0.24* (-0.39, 0.59)	1.83	5.06 (25.3%)
Estimation discrepancy-DR	0.59* (0.26, 0.78)	1.18	3.28 (16.41%)	0.39* (-0.11, 0.67)	1.81	5.03 (25.14%)
Self-perception and evaluation						
Perceives task as difficult-IR	0.78 (0.59, 0.88)	0.67	1.86 (15.54%)	0.92 (0.86, 0.96)	0.34	0.96 (7.96%)
Perceives task as difficult-DR	0.84 (0.71, 0.91)	0.55	1.52 (12.65%)	0.84 (0.71, 0.92)	0.53	1.46 (12.19%)
Immediate recall (IR)	0.79 (0.62, 0.89)	1.09	3.28 (15.13%)	0.67 (0.39. 0.82)	1.24	3.45 (17.24%)
Delayed recall (DR)	0.74 (0.52, 0.86)	1.37	3.79 (18.97%)	0.69 (0.42, 0.83)	1.82	5.06 (25.28%)
Total recall (TR)	0.81 (0.66, 0.9)	2.05	5.69 (14.24%)	0.72 (0.5, 0.85)	2.75	7.63 (19.08%)
Total strategy use (TSS)	0.63 (0.31, 0.8)	1.06	2.94 (24.54%)	0.41* (-0.08, 0.68)	1.53	4.23 (35.24%)*
General awareness	0.68 (0.41, 0.83)	0.95	2.63 (9.05%)			
Recognition-morning	0.85 (0.71, 0.92)	0.39	1.08 (5.44%)			
Recognition-restaurant	0.76 (0.56, 0.87)	0.52	1.45 (7.26%)			

ICC, intraclass correlation coefficient; CI, confidence interval; SEM, standard error of measurement; MDC_95_, the minimal detectable change calculated based on 95% confidence interval.

*ICC< 0.6 or MDC % >30%.

Table 3 summarizes the ICC, SEM and MDC values of all CMT domains in Part I, Part II and the recognition subtest in MCI participants. Most ICC values were between 0.73 and 0.94, indicating good to excellent reliability except the self-efficacy domain (the estimation discrepancy, Part I and II) and TSS domain (Part II). The ICC values of the self-efficacy and TSS domains were below 0.6 (range: 0.07–0.48). The SEM values were between 0.42 and 2.12. Only MDC% values of TSS domain (Part II) were above 30% (35.14%).

**Table 3:** Results of the test-retest reliability and MDC of the CMT in MCI participants (n = 39).

	Part I. No context cue			Part II. Context cue		
	ICC (95% CI)	SEM	MDC_95_ (MDC%)	ICC (95% CI)	SEM	MDC_95_ (MDC%)
Self-efficacy						
Prediction discrepancy	0.73 (0.48, 0.86)	2.11	5.84 (29.21%)			
Estimation discrepancy-IR	0.46* (-0.03, 0.72)	1.98	5.47 (27.37%)	0.07* (-0.76, 0.51)	1.58	4.38 (21.89%)
Estimation discrepancy-DR	0.42* (-0.11, 0.7)	2.08	5.77 (28.83%)	0.21* (-0.49, 0.58)	2.01	5.57 (27.83%)
Self-perception and evaluation						
Perceives task as difficult-IR	0.93 (0.86, 0.96)	0.55	1.53 (12.76%)	0.94 (0.88, 0.97)	0.42	1.17 (9.76%)
Perceives task as difficult-DR	0.9 (0.81, 0.95)	0.66	1.83 (15.22%)	0.91 (0.83, 0.95)	0.55	1.54 (12.8%)
Immediate recall (IR)	0.91 (0.83, 0.95)	1.08	3 (15.01%)	0.88 (0.77, 0.94)	1.1	3.04 (15.18%)
Delayed recall (DR)	0.87 (0.75, 0.93)	1.36	3.79 (18.93%)	0.91 (0.82, 0.95)	1.03	2.87 (14.33%)
Total recall (TR)	0.91 (0.84, 0.95)	2.12	5.88 (14.7%)	0.91 (0.86, 0.96)	1.9	5.28 (13.19%)
Total strategy use (TSS)	0.76 (0.54, 0.87)	0.93	2.59 (21.54%)	0.48* (0.01,0.73)	1.52	4.22 (35.14%)*
General awareness	0.93 (0.87,0.96)	0.75	2.08 (7.17%)			
Recognition-morning	0.84 (0.7, 0.92)	1.14	3.16 (15.79%)			
Recognition- restaurant	0.78 (0.57, 0.88)	1.38	3.83 (19.13%)			

ICC, intraclass correlation coefficient; CI, confidence interval; SEM, standard error of measurement; MDC_95_, the minimal detectable change calculated based on 95% confidence interval; MCI, mild cognitive impairment.

*ICC<0.6 or MDC %>30%.

Table 4 shows the Bland-Altman analysis results. The MD of the two measurements significantly deviated from 0 in most CMT domains, including the self-efficacy (estimation discrepancy, healthy, *P* = 0.03; prediction discrepancy, MCI, *P* = 0.01), self-perception and evaluation (MCI, *P* <0.001–0.007), IR, DR, TR (*P*<0.001), TSS (MCI, *P*<0.001–0.05) and the recognition subtest (healthy, morning, *P* = 0.02; MCI, restaurant, *P* = 0.004). Less prediction/estimation discrepancy, reduction of feeling of task as difficult and greater recall, recognition and TSS scores were found in the 2^nd^ than the 1^st^ measurement in healthy and MCI participants.

**Table 4:** Results of the Bland-Altman analysis in healthy and MCI participants.

	Part I. No context cue						Part II. Context cue					
	Healthy			MCI			Healthy			MCI		
	MD (SD)	95% LOA	*P*	MD (SD)	95% LOA	*P*	MD (SD)	95% LOA	*P*	MD (SD)	95% LOA	*P*
Self-efficacy												
Prediction discrepancy	-1 (3.64)	6.15, -8.15	0.08	-1.58 (3.67)	5.61, -8.79	0.01*						
Estimation discrepancy-IR	-0.23 (3)	5.66, -6.11	0.62	-0.58 (3.1)	5.49, -6.67	0.24	-0.38 (2.68)	4.88, -5.65	0.35	-0.1 (2.76)	5.31, -5.51	0.82
Estimation discrepancy-DR	-0.77 (2.27)	3.68, -5.22	0.03*	-0.44 (3.04)	5.53, -6.4	0.38	-0.45 (2.67)	4.78, -5.69	0.27	-0.97 (3.51)	5.91, -7.86	0.09
Self-perception and evaluation												
Perceives task as difficult-IR	-0.23 (1.16)	2.04, -2.5	0.2	-0.67 (1.01)	1.31, -2.64	<0.001*	-0.14 (0.63)	1.1, -1.38	0.16	-0.38 (0.85)	1.27, -2.04	0.007*
Perceives task as difficult-DR	-0.36 (0.99)	1.58, -2.3	0.2	-0.56 (1.16)	1.72, -2.85	0.004*	-0.18 (0.92)	1.63, -1.99	0.2	-0.26 (1.01)	1.64, -2.25	0.12
Immediate recall (IR)	1.59 (1.83)	5.19, -2	<0.001*	2.05 (1.99)	5.97, -1.87	<0.001*	1.59 (2.06)	5.63, -2.45	<0.001*	1.46 (2.28)	5.91, -3.01	<0.001*
Delayed recall (DR)	2.2 (2.28)	6.67, -2.26	<0.001*	1.64 (2.64)	6.82, -3.53	<0.001*	1.15 (2.76)	6.57, -4.25	<0.001*	1.51 (2.21)	5.85, -2.82	<0.001*
Total recall (TR)	3.79 (3.46)	10.59, -3	<0.001*	3.69 (4.01)	11.55, -4.16	<0.001*	2.75 (4.33)	11.24, -5.74	<0.001*	2.97 (3.71)	10.26,- 4.31	<0.001*
Total strategy use (TSS)	-0.23 (1.66)	3.04, -3.5	0.37	0.33 (1.57)	3.43, -2.76	0.2	0.45 (4.33)	4.61, -3.7	<0.001*	0.74 (2.28)	5.21, -3.72	0.05*
General awareness	0.18(1.74)	3.6, -3.23	0.49	0.15 (1.46)	3.02, -2.71	0.52						
Recognition-morning	0.26 (0.66)	1.56, -1.04	0.02*	0.47 (2.23)	4.86, -3.91	0.2						
Recognition-restaurant	0.22 (0.79)	1.77, -1.33	0.08	1.23 (2.48)	6.11, -3.64	0.004*						

MD, mean differences, the values of the 2^nd^ test subtract the value of the 1^st^ test; SD, standard deviation; LOA, limits of agreement; MCI, mild cognitive impairment.

*P<0.05.

## Discussion

To the best of our knowledge, this is the first study to comprehensively examine the test-retest reliability and MDC of all CMT domains in healthy and MCI older adults. We found that memory and meta-memory function can be reliably assessed by most of the CMT domains in healthy and MCI participants except the TSS (Part II) and self-efficacy (Part I and II) domains. The ICC value was low and the MDC% was large in the TSS and self-efficacy domains in both healthy and MCI participants. The educational level was higher in healthy than MCI participants. Our study expanded findings of previous studies by showing that memory and meta-memory performance can be reliably assessed by most CMT domains in older adults with and without cognitive impairment. The ICC values reported in our study were also comparable to those of previous studies in individuals with neurological impairment (ICC = 0.73–0.81) [16–18]. This result indicates that the CMT can be used to assess memory and meta-memory function in not only individuals with brain injuries but also those with mild cognitive impairment.

Furthermore, we identified similar patterns of responses in the healthy and MCI participants, where the low test-retest reliability (i.e., ICC value) and large measurement errors (i.e., MDC%) were found in the TSS (Part II) and self-efficacy domains (Part I & II). This result indicated that use of memory strategies and self-efficacy might not be reliably assessed by the CMT in older adults irrespective of their educational levels or whether they had cognitive impairment. Two possible reasons may explain the insufficient test-retest reliability of these two domains. First, scoring of TSS in the context cue condition depended on the types of strategies adopted by the participants. The scores would be higher if participants used strategies fully or partially related to the contexts (i.e., morning/restaurant). Nevertheless, studies have shown that the familiarity to the contexts determined if episodic memory strategies would be used [33, 34]. If individuals are familiar with the association of items and contexts, they will have greater chances to trigger episodic memory and used context cues as the primary strategy to recall items [35, 36]. It may be possible that some of the items on the morning/restaurant picture cards are not embedded in participants’ usual morning /restaurant routines; consequently, providing context cues to these participants may not necessarily activate their episodic memory related to the morning/restaurant contexts and facilitate contextual strategy use. Instead, these participants may adopt various types of memory strategies, such as the sematic memory strategy (i.e., remembering the facts of the items), location or visualization of items, and caused high variances in measurements [37].

Similarly, self-efficacy domains including the prediction and estimation of performance may also rely on the prior knowledge of the contexts [38–40]. Participants may predict or estimate their performance more precisely and consistently if items on the picture cards are involved in their usual morning/restaurant routines and already built in their episodic memory [39]. In contrast, participants’ prediction or estimation performance may be less stable with greater random errors when they are unfamiliar with the association between the items and morning/restaurant contexts, thus resulting in a higher MDC value.

To minimize the potential impact of unfamiliarity of items/contexts on assessing strategy-use, we recommend expansion of current items on the morning/restaurant cards and individually select items that are relevant to each individual’s morning/restaurant routines [41]. For example, evaluators could ask individuals about their morning/restaurant routines to determine which items should be included in the picture card test. This will ensure that the items to remember/recall are coherent with the contexts, thus enabling true evaluation of individuals’ ability to utilize contextual strategies [42]. In addition, we recommended adding questions to examine the degrees of familiarity of items/contexts to the participants [39]. Questions such as “How familiar were these items to you?” or “Were these items involved in your usual morning/restaurant routines?” can be added to the CMT to determine if individuals’ strategy use and self-efficacy are affected by the unfamiliarity or solely by their memory/meta-memory dysfunction [43, 44]. Future studies could evaluate the impact of familiarity of activities of daily living items/contexts on contextual strategy uses and determine if individualization of items/contexts can help to improve test-retest reliability in the TSS and self-efficacy domains.

Second, it may also be possible that participants may have used contextual strategies but were unware of it or unable to verbalize it, which may affect TSS scores and result in higher variability and unstable measurements [45, 46]. To minimize this potential confounding effect, we recommended evaluators to analyze the order of recalled items along with the TSS scores to determine if participants had used contextual strategies. This will ensure that the TSS domain was scored appropriately.

We also identified large MDC% in the TSS and self-efficacy domains, indicating potentially greater random errors in these two domains. The relatively greater random errors in these two domains could be due to the inherent dynamic feature of meta-memory monitoring process. The meta-memory monitoring process, such as prediction or estimation of current memory performance is a dynamic process and may be affected by daily events and memory/meta-memory state of each person [47, 48]. Studies have also shown that day-to-day fluctuation of arousal, mental or environmental demands affected participants’ sense of control over their memory performance and the strategy they use to compensate for the unstable memory performance [48, 49]. Thus, it may be plausible that the fluctuated sense of control over memory performance led to the random errors in the self-efficacy and TSS domains and caused large MDC%. To prevent misinterpretation of CMT results in older adults, the MDC values reported in this study can be considered as a reference to determine if the observed changes overtime reflect real performance changes. In addition, we also recommend assessing these two domains at the same time of the day (e.g., morning or afternoon) in the same environment to minimize the influence of arousal levels/environments on measurements [47].

In addition to the assessment of ICC values, we also performed the Bland-Altman analysis to evaluate the agreement between two measurements. We found generally better performance (i.e., higher scores and fewer prediction/estimation errors) in the 2^nd^ than the 1^st^ measurement in most CMT domains in both groups of participants. This finding indicates that there may be potentially practice effects of CMT in healthy and MCI participants [50, 51]. Participants may have remembered the items/contexts or learned the contextual strategy in the 1^st^ test and therefore improved their memory and meta-memory performance in the 2^nd^ test. This result was consistent with the findings of practice effects of cognitive tests on learning and memory in the literatures for healthy and cognitively-impaired adults [51, 52]. To minimize the potential practice effects of CMT, we recommended using the dual-baseline approach. The dual-baseline approach has been suggested to be a useful approach to reduce practice effects of cognitive measurements [53, 54]. The CMT test can be administered twice in the same person within a few days or weeks. If significantly prominent improvement occurs from the 1^st^ to the 2^nd^ measurement, the 2^nd^ measurement may serve as the baseline for subsequent analysis [50, 55]. Future studies could examine the best appropriate interval of two assessments for minimizing practice effects.

Seven limitations should be considered. First, although we have demonstrated that the CMT is appropriate for assessing memory and meta-memory in healthy and MCI participants, the usability of CMT in older adults with more severe cognitive impairment, such as dementia remains unknown. Second, we did not evaluate the validity of CMT, such as the concurrent validity in older adults although it has been established in individuals with brain injuries [16]. Future studies could include moderate-to-severe cognitively impaired older adults and examine the concurrent validity of CMT by comparing the CMT to other standardized memory/meta-memory assessments. Third, the cutoff value of the MDC% (i.e., 30%) was selected based on the recommendation in the literatures [28, 29]. However, we also provided the raw value of the MDC and the MDC% of each CMT domain. Future studies could use these raw values to determine their own cutoff points based on the study purposes. Fourth, the sample size of the study was relatively small although it was comparable to that of previous CMT studies [16–18]. Future study could recruit a larger sample of healthy and MCI participants to validate findings of this study. Fifth, we used the MOCA to identify whether participants had MCI; however, we did not assess the potential etiology of MCI using other assessments such as neurophysiological tests or brain imaging (e.g., Magnetic resonance imaging, MRI). Future study could examine the etiology of MCI participants to determine whether different etiology of MCI would affect the test-retest reliability of the CMT in these individuals. Sixth, the educational level was higher in the healthy than MCI participants although similar patterns of responses (i.e., low ICC and large MDC% of the TSS and self-efficacy domain) were found between the two groups. Future study could recruit MCI participants with different educational levels (e.g., high and low) to determine if the educational level would affect the test-retest reliability of CMT in MCI participants. Seventh, participants were evaluated in a quiet room and were required to relax during the break between the immediate and delayed recall condition as well as between Part I and Part II. This was to minimize the potential interference from the environment or conditions. Our results also showed good test-retest reliability in the immediate and delayed recall CMT domains, indicating that the procedures we performed may be useful. However, it is still possible that there were other environmental/conditional interferences that we did not control. Future studies are warranted to determine the best testing environments/conditions for performing the CMT in healthy and MCI participants.

## Conclusions

Most memory and meta-memory tests do not directly assess memory/meta-memory performance related to daily contexts [7–13]. The CMT is specifically designed for evaluating awareness, self-perception and evaluation, self-efficacy, recall and strategy-use involving common daily contexts and therefore can be a potentially useful screening tool for identifying individuals that may have memory or meta-memory impairment in daily contexts and an assessment tool for evaluating memory and meta-memory changes involving daily tasks after treatments [16–18]. The CMT can also be used in conjunction with other memory or meta-memory assessments to provide a comprehensive evaluation of individuals’ cognitive ability related to activities of daily living.

Our study revealed that most CMT domains had sufficient test-retest reliability in healthy and MCI older adults. Only two domains (i.e., TSS and self-efficacy) had low test-retest reliability and large random errors, which could be due to participants’ unfamiliarity with the association between the items and contexts and the dynamic feature of meta-memory. We also identified potential practice effects of CMT in healthy and MCI participants. Clinicians/therapists should be cautious when explaining CMT scores between repeated measurements. Future studies are warranted to examine if individual selection of items based on each person’s familiar morning/restaurant routines along with the dual-baseline approach could help to improve test-retest reliability and reduce measurement errors of the CMT. This will enhance the usability of CMT in diagnosis of memory/meta-memory impairment and prognosis of memory/meta-memory recovery associated with daily contexts in the elderly.

## Supporting information

S1 File The Bland-Altman plots of all domains (Part I, Part II and Recognition Subtest) of the CMT in healthy and MCI participants. (DOCX)
