# Supplementary material for: Test-retest reliability and minimal detectable change of the Contextual Memory Test in older adults with and without mild cognitive impairment
Source: PLoS One. 2020 Jul 31;15(7):e0236654. doi: 10.1371/journal.pone.0236654 (PMC7394426; doi:10.1371/journal.pone.0236654)
Supplement: S1 File — (DOCX) [file pone.0236654.s001.docx]

**S1. The Bland-Altman Plots of the CMT(Part I, Part II and Recognition Subtest) in healthy and MCI participants**

Supplementary figure 1: Bland-Altman Plots of Part I in healthy participants


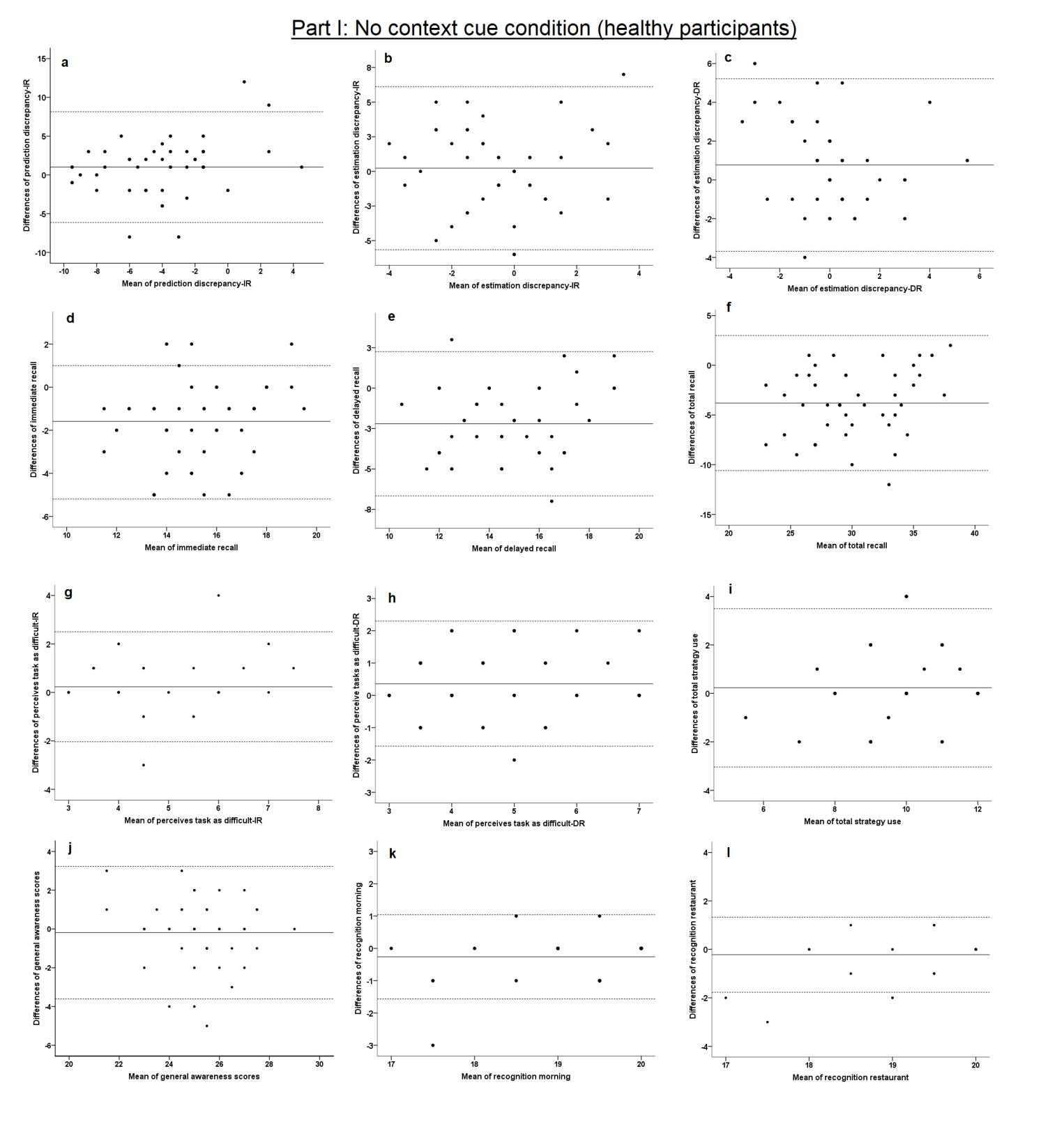
(a) Prediction discrepancy-immediate recall; (b) Estimation discrepancy-immediate recall; (c) Estimation discrepancy-delayed recall; (d) Immediate recall; (e) Delayed recall; (f) Total recall; (g) Perceive task as difficult-immediate recall; (h) Perceive task as difficult-delayed recall; (i) Total strategy use; (j) General awareness; (k) Recognition subtest-morning; (l) Recognition subtest-restaurant. The bold solid line is the mean difference. The two dotted lines are the 95% limits of agreement.


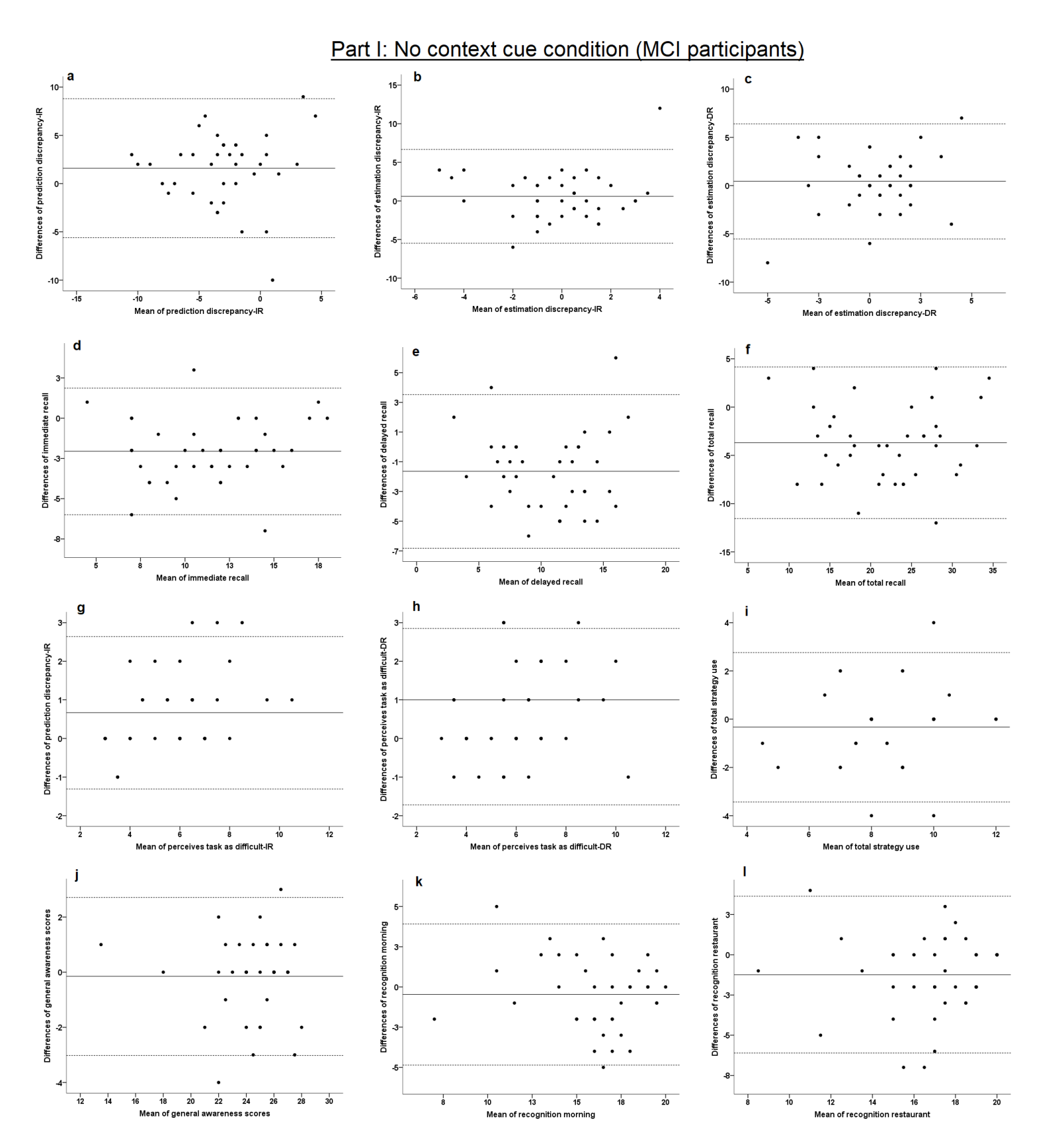
Supplementary figure 2: Bland-Altman Plots of Part I in MCI participants

(a) Prediction discrepancy-immediate recall; (b) Estimation discrepancy-immediate recall; (c) Estimation discrepancy-delayed recall; (d) Immediate recall; (e) Delayed recall; (f) Total recall; (g) Perceive task as difficult-immediate recall; (h) Perceive task as difficult-delayed recall; (i) Total strategy use; (j) General awareness; (k) Recognition subtest-morning; (l) Recognition subtest-restaurant. The bold solid line is the mean difference. The two dotted lines are the 95% limits of agreement.


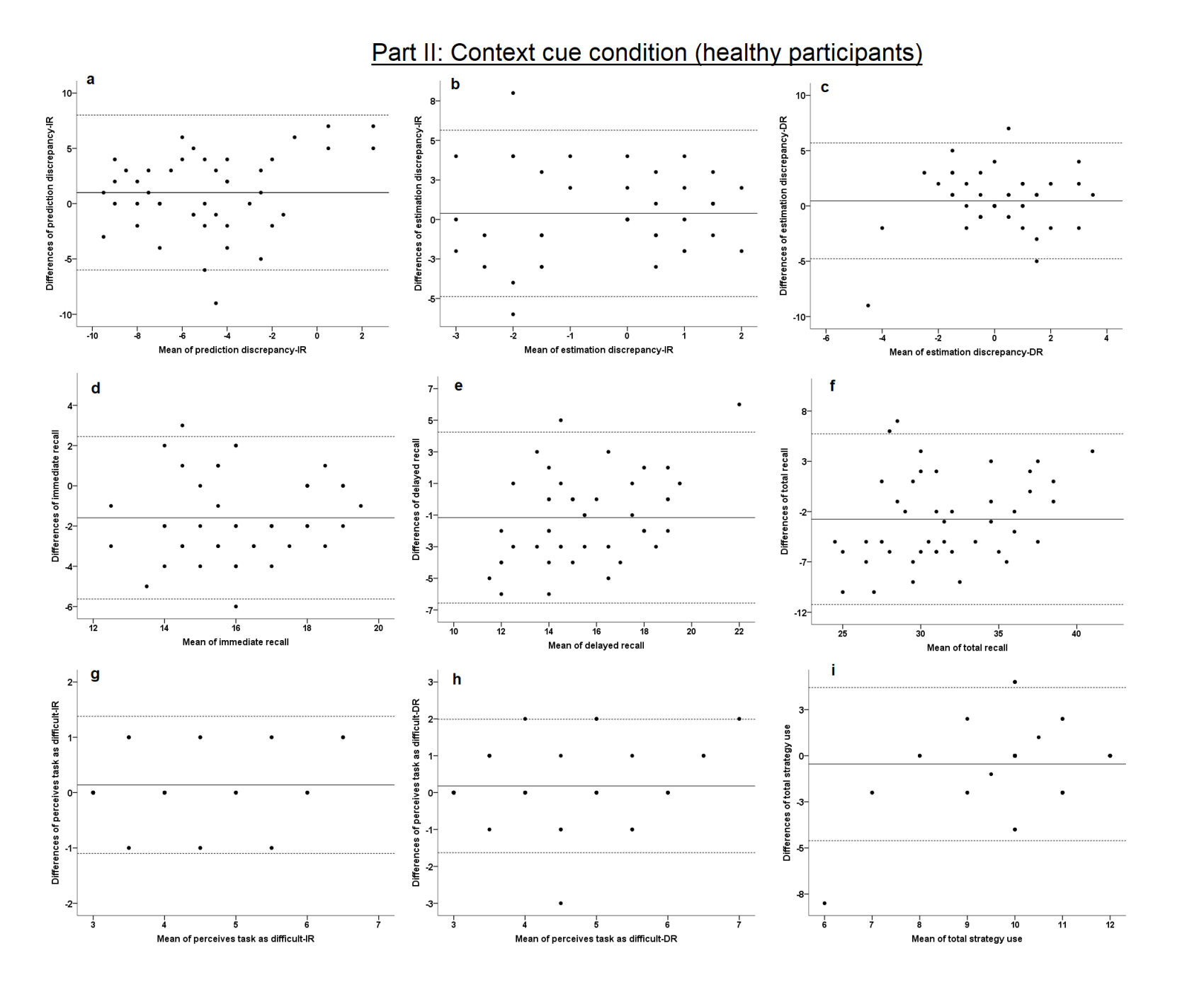
Supplementary figure 3: Bland-Altman Plots of Part II in healthy participants

(a) Prediction discrepancy-immediate recall; (b) Estimation discrepancy-immediate recall; (c) Estimation discrepancy-delayed recall; (d) Immediate recall; (e) Delayed recall; (f) Total recall; (g) Perceive task as difficult-immediate recall; (h) Perceive task as difficult-delayed recall; (i) Total strategy use. The bold solid line is the mean difference. The two dotted lines are the 95% limits of agreement.


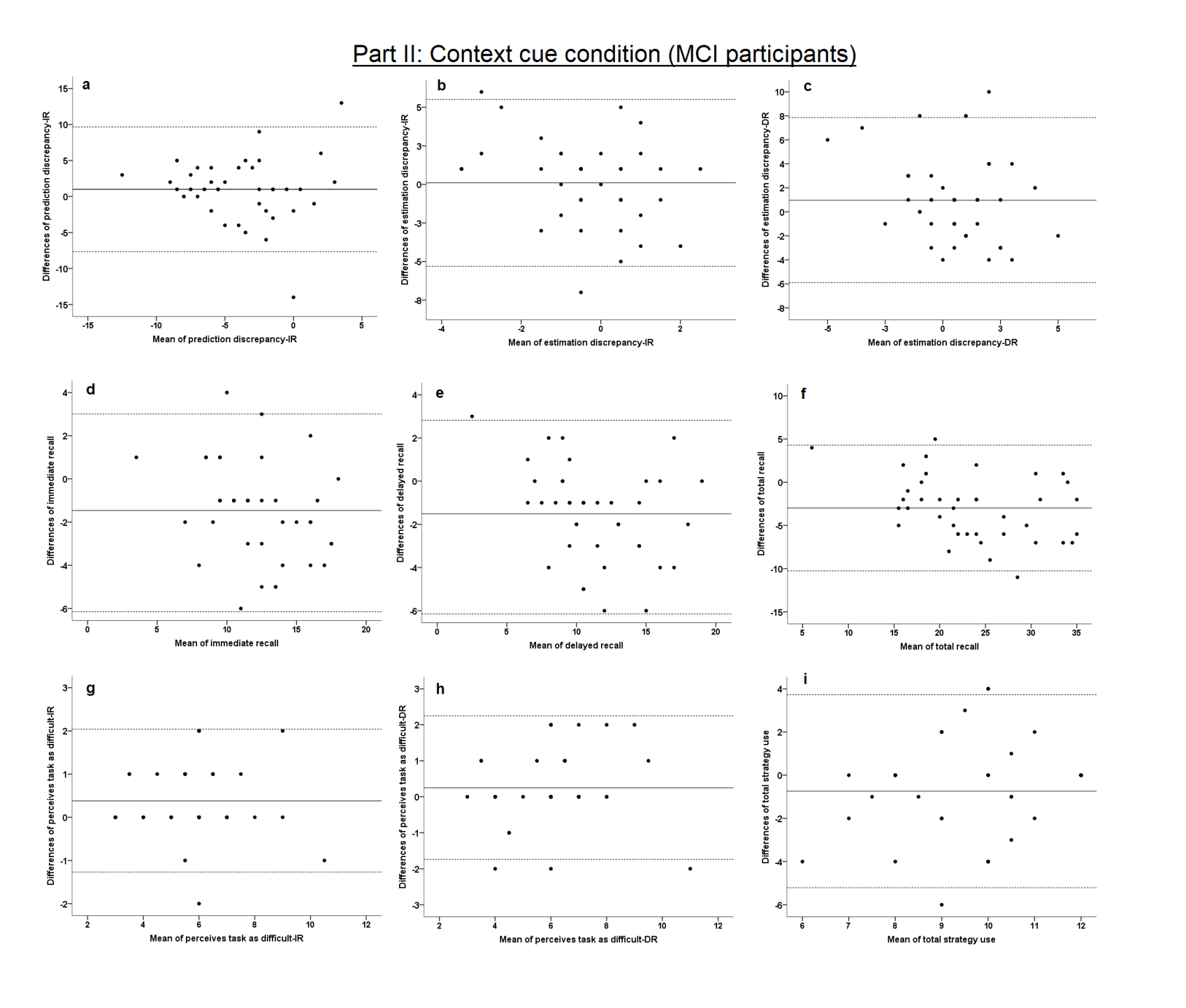
Supplementary figure 4: Bland-Altman Plots of Part II in MCI participants

(a) Prediction discrepancy-immediate recall; (b) Estimation discrepancy-immediate recall; (c) Estimation discrepancy-delayed recall; (d) Immediate recall; (e) Delayed recall; (f) Total recall; (g) Perceive task as difficult-immediate recall; (h) Perceive task as difficult-delayed recall; (i) Total strategy use. The bold solid line is the mean difference. The two dotted lines are the 95% limits of agreement.
